# Pedestrian-Accessible Infrastructure Inventory: Enabling and Assessing Zero-Shot Segmentation on Multi-Mode Geospatial Data for All Pedestrian Types

**DOI:** 10.3390/jimaging10030052

**Published:** 2024-02-21

**Authors:** Jiahao Xia, Gavin Gong, Jiawei Liu, Zhigang Zhu, Hao Tang

**Affiliations:** 1 Department of Civil and Environmental Engineering, Rutgers, The State University of New Jersey, Piscataway, NJ 08854, USA; jx198@soe.rutgers.edu; 2 East Brunswick High School, East Brunswick, NJ 08816, USA; gonggavineb@gmail.com; 3 Department of Computer Science, The CUNY Graduate Center, New York, NY 10016, USA; jliu9@gradcenter.cuny.edu; 4 Department of Computer Science, The CUNY City College and The CUNY Graduate Center, New York, NY 10031, USA; zzhu@ccny.cuny.edu; 5 Department of Computer Information Systems, The CUNY BMCC and The CUNY Graduate Center, New York, NY 10007, USA

**Keywords:** pedestrian infrastructure, multi-sourced, geospatial data, deep learning, zero-shot method, computer vision, visually impaired

## Abstract

In this paper, a Segment Anything Model (SAM)-based pedestrian infrastructure segmentation workflow is designed and optimized, which is capable of efficiently processing multi-sourced geospatial data, including LiDAR data and satellite imagery data. We used an expanded definition of pedestrian infrastructure inventory, which goes beyond the traditional transportation elements to include street furniture objects that are important for accessibility but are often omitted from the traditional definition. Our contributions lie in producing the necessary knowledge to answer the following three questions. First, how can mobile LiDAR technology be leveraged to produce comprehensive pedestrian-accessible infrastructure inventory? Second, which data representation can facilitate zero-shot segmentation of infrastructure objects with SAM? Third, how well does the SAM-based method perform on segmenting pedestrian infrastructure objects? Our proposed method is designed to efficiently create pedestrian-accessible infrastructure inventory through the zero-shot segmentation of multi-sourced geospatial datasets. Through addressing three research questions, we show how the multi-mode data should be prepared, what data representation works best for what asset features, and how SAM performs on these data presentations. Our findings indicate that street-view images generated from mobile LiDAR point-cloud data, when paired with satellite imagery data, can work efficiently with SAM to create a scalable pedestrian infrastructure inventory approach with immediate benefits to GIS professionals, city managers, transportation owners, and walkers, especially those with travel-limiting disabilities, such as individuals who are blind, have low vision, or experience mobility disabilities.

## 1. Introduction

Walkability is perhaps the biggest defining characteristic of a healthy and equitable community, as recent literature points to numerous benefits associated with communities with greater walkability [1]. These benefits include, but are not limited to, the reduction of obesity and cardiovascular illness, improved physical well-being, and even a reduction of depression symptoms [2]. This is especially true for those with travel-limiting disabilities, such as individuals who are blind, have low vision, or experience mobility disabilities. Historically, the development of large urban centers has been biased towards serving a car-dominant culture, inevitably sacrificing walkability, increasing congestion and air pollution in urban centers, and undermining walking as an enjoyable journey in these communities. In our constantly changing climate, the unprecedented number of natural events, such as heatwaves and wildfires, is also impacting the walkability of communities. It appears that we are being caught in an irreversible downward spiral, a trend where growing car-centric designs and industries are encouraging driving over walking. In fact, these carbon-based automobile industries represent one of the biggest causes of the decline of walkability as they also identify as one of the largest stimuli of climate change, a phenomenon which increases the frequency of extreme weather events and dampens the public’s desire to walk [3]. To combat this downward trend, we need to make significant changes to pedestrian infrastructures in communities so that they can provide convenient, safe, and enjoyable walking experiences for everyone, especially those with visual and mobility disabilities.

Traditionally, the term ‘pedestrian infrastructure’ refers to a set of transportation elements such as sidewalks, trails, crosswalks, and intersection designs. Expanding this definition with accessibility features allows us to include amenities along the roads, public arts, and wayfinding services to make walking a truly enjoyable experience for all, including people with disabilities. Further expansion of this definition allows for the inclusion of variations of street landscapes, such as tree canopies and street furniture, to address thermal comfort and physical fatigue [3]. These expanded definitions are closely tied to the aspiration to serve all pedestrian types, especially people facing challenges such as those with visual and mobility disabilities, in rehabilitation, or who are elderly [4]. One major result of this aspiration is an increasing demand for the digitization of current urban environments with a greater level of detail so that the state of the pedestrian infrastructure in a community can be assessed with higher confidence.

*Pedestrian infrastructure inventory* is a common means utilized by transportation agencies to assess the state of pedestrian infrastructure and to determine maintenance and improvement needs. There is already a rich body of literature on various methods used for creating pedestrian infrastructure inventory. A significant portion of the work in this domain relied on field observations [5,6] and street-view photo-based analysis [7]. Remote sensing technologies enabled the utilization of satellite images and point-cloud data for digitizing pedestrian infrastructure [8,9,10,11,12,13,14,15]. Among these technologies, vehicular mapping systems such as mobile LiDAR have recently gained increased attention from the transportation community due to their capability to collect accurate and dense urban point-cloud sets at street level. The rise of social media also added a dimension of data collection through crowdsourcing and volunteered geographic information (VGI) [16,17]. However, it is important to recognize that even with these notable advancements, none of the data collection methods cited above can truly create a complete picture of the pedestrian infrastructure in a community. Particularly when adopting and defining the term ‘pedestrian infrastructure’ in a broader state, it becomes quite apparent that the strengths of each inventory method are balanced out by their weaknesses. There is a lack of understanding of the potential of various geospatial technologies, especially the recent ones such as mobile LiDAR, to fulfill the needs of creating pedestrian-accessible infrastructure inventory. This recognition has led to studies exploring the fusion of multiple types of data for pedestrian infrastructure inventory [18,19,20,21].

With the increasing amount of data that can be collected on pedestrian infrastructures, computer vision and machine-learning methods have become mainstream research due to the challenge of manually processing these large datasets. Despite numerous proposed computer vision and machine-learning models for pedestrian infrastructure inventory, in particular, for accessible sidewalk inventory, the new categories of pedestrian infrastructure being considered by equity-minded researchers present significant challenges to existing pedestrian inventory practices and research, a consequence of the unlikelihood that data from single sources and AI models trained for extracting specific features would be able to provide adequate information on all pedestrian infrastructure features. There is an endless need for more and new annotated data for pedestrian infrastructure objects. Luckily, foundation models that can generalize to data that they have not seen are considerably successful in tasks including natural language process (NLP) and computer vision [22]. The Segment Anything Model (SAM) [23], released by Meta, is one of the foundation models for image segmentation, showing remarkable performance in zero-shot learning. In the context of pedestrian infrastructure inventory, SAM has great but unexplored potentials that could significantly reduce the need for labeling a large amount of data and, therefore, improve the generalizability of trained computer vision models.

In this project, we aim to develop a novel approach that can process multi-mode geospatial datasets, including mobile LiDAR and satellite imagery, using the Segment Anything Model (SAM), to generate comprehensive ***pedestrian-accessible infrastructure*** databases for all pedestrian types. Throughout our project, we intend to address three entwined questions:

(1) How can mobile LiDAR technology be leveraged to produce comprehensive pedestrian-accessible infrastructure inventory?
(2) How well does SAM generalize to mobile LiDAR and satellite imagery data in representative segmentation tasks for pedestrian-accessible infrastructure inventory work?
(3) What data representations can effectively boost zero-shot image segmentation performance with SAM for pedestrian-accessible infrastructure inventory work?

The pedestrian-accessible infrastructure features considered in this paper are not only inclusive of **traditional features** such as *sidewalks*, *crosswalks*, *detectable warning surfaces*, and *curb ramps*, but are also inclusive of numerous types of **street furniture**, particularly those impacting walkability and accessibility. These include but are not limited to: *benches*; *bollards*; *fire hydrants*; *landscapes*; *mailboxes*; *manhole covers*; *memorials*; *phone booths*; *parking meters*; *posts*; *public sculptures*; *public vending machines*; *stairs*; *storm water inlets*; *traffic barriers*; *trees*; and *waste containers*. Each of the objects mentioned above certainly has the potential to hinder navigation as they can occupy space on the sidewalks and pose obstacles that can be particularly treacherous for disabled people. However, through the utilization of their locations on the sidewalks, the objects can also assist in navigation by serving as landmarks and indicators for walkers.

The proposed method can extract the accurate 3D position of all these obstacles, which are omitted in previous studies, and it can be used as high-definition maps and support the navigation for individuals with visual impairments. Our proposed method is designed to efficiently create pedestrian-accessible infrastructure inventory through the zero-shot segmentation of multi-sourced geospatial datasets. Through addressing three research questions, we show how the multi-mode data should be prepared, what data representation works best for what asset features, and how SAM performs on these data presentations. The findings also show what needs to be done to improve the proposed workflow for further scalable extraction of pedestrian-accessible infrastructure inventory.

## 2. Literature Review

As an indispensable infrastructure, sidewalks support the essential daily trips of pedestrians, especially those with travel-limiting disabilities. Developing a comprehensive sidewalk inventory attracts increasing interest among researchers from various fields, such as urban studies and urban mobility. Based on the data sources, we classified sidewalk-related studies into three categories: (1) image-based, (2) point-cloud-based, and (3) data fusion-based.

### 2.1. Image-Based

Most image-based studies focus on localizing the sidewalks using satellite imagery or geo-tagged street-level images. Luo, Wu [11] extracted connected sidewalk network from aerial images and the common occlusion problem resulting from trees and their shadows can bring large uncertainty to the detected sidewalks under the bird’s eye view [12]. Hosseini et al. [10] developed a scalable satellite imagery-based sidewalk inventory method. Street-view images are another data source used for assessing sidewalk accessibilities. Rao et al. [24] developed computer vision methods to detect potholes and uneven surfaces on sidewalks to assist blind people. Li et al. [25] developed Presight, a system capable of detecting sidewalk accessibility problems in real time during trips. Many recent studies also explored the use of large open geospatial datasets, particularly Google Street View (GSV), to create sidewalk accessibility databases [7,21]. A common issue about these image-based methods is that detailed geometry attributes of the sidewalks, such as grades and cross slopes, are beyond the scope of 2D optical images, considering their limited spatial accuracy. However, these geometric features are essential to accessibility analysis for those who are visually impaired or who rely heavily on wheelchairs, canes, crutches, or walkers [26].

### 2.2. Point-Cloud-Based

Recently, Light Detection and Ranging (LiDAR) technology has been increasingly used to create infrastructure inventory. Horváth, Pozna [15] detected sidewalk edge in real time using point clouds collected by autonomous driving cars. Hou and Ai [14] extracted sidewalks through segmenting point clouds using deep neural networks. Esmor et al. [27] used mobile LiDAR data to characterize zebra cross zones. However, these typical supervised learning-based 3D segmentations require large, annotated training datasets, and creating these datasets is highly time-consuming and labor-intensive.

### 2.3. Data Fusion-Based

Recognizing the limitations of a single type of data collection method, researchers have also studied the use of multi-modal data to improve the model performance. Luaces et al. [13] studied the fusion of LiDAR data and social media data for sidewalk inventory. Ai and Tsai [13] extracted sidewalks and curb ramps using both point clouds and video frames. However, in most data fusion-based research, some factors, such as the misalignment of different sensors, can adversely affect the results of multi-modal fusion.

Scaling up the training database to include more categories of pedestrian infrastructure objects, such as those described in the introduction section, is a common challenge in existing pedestrian infrastructure inventory research. Designing a framework with strong generalization ability has been a long-sought research goal. The Segment Anything Model (SAM) is a foundation model for image segmentation tasks [23]. SAM’s support for zero-shot image segmentation with various prompts such as points, boxes, and masks makes it highly attractive for image segmentation tasks in pedestrian infrastructure inventory work. However, there is currently no assessment of SAM-based zero-shot segmentation on data used in pedestrian infrastructure inventory. This leads to the present project, which aims to evaluate the performance of SAM in segmenting a broad range of pedestrian infrastructure elements from multi-sourced geospatial datasets.

## 3. Methodology

### 3.1. Description of Data Collection Methodology and Datasets

Satellite imagery and 3D point-cloud data are among the common types of data that have been studied for extracting pedestrian infrastructure features at the network level. In our project, we use mobile LiDAR point-cloud data as the primary data source, and bring in Google satellite imagery as the secondary data source. The point cloud encompasses various features, including precise 3D coordinates, GPS time, intensity, and color. Notably, intensity and color information play a pivotal role in facilitating the extraction of semantic information.

The mobile LiDAR system used in this study is equipped with an Inertia aided Global Navigation Satellite System (GNSS), a Z + F 9012 profiler, and a Z + F mapcam. The GNSS system includes a tactile grade Inertia Measurement Unit (IMU) with 200 HZ frequency and dual Trimble GPS antennas. The system is capable of computing accurate vehicle trajectories in challenging urban environments. The Z + F profiler can collect as many as 2 million points per second. The Z + F mapcam consists of five 10-megapixel cameras that can record street views at 2–10 frames per second at driving speed. Both the profiler and mapcam are triggered and synchronized by the precise GPS Pulse Per Second (PPS) signal to ensure that they have consistent global time stamps to align their sensor readings. Both systems are installed with known lever arms to the IMU and precisely calibrated to the center of the IMU. With highly accurate time synchronization and calibration, the point-cloud data from the laser profiler and the street-view imagery from the mapcam can be aligned and co-registered to a global coordinate system. For each data collection trip, additional calibration steps such as bore-sight are also conducted to compensate for changes in sensor alignment due to factors such as tire pressure change and small movement of sensor installations during use. Therefore, compared to data from social media outlets or data from typical mapping systems used on autonomous driving vehicles, point-cloud and imagery data from a survey-grade mapping system have the advantage of not only being highly accurate but also having more precise alignments among themselves than other non-survey-grade systems. This advantage allows the convenient fusion of features extracted from both datasets. However, the sensor misalignment issue cannot be eliminated in any mapping system. When co-registered color images are used to colorize point-cloud data, there is inevitable blurring of edges. One of the interests of this study is to see how these artifacts influence segmentation results.

**Figure 1:**
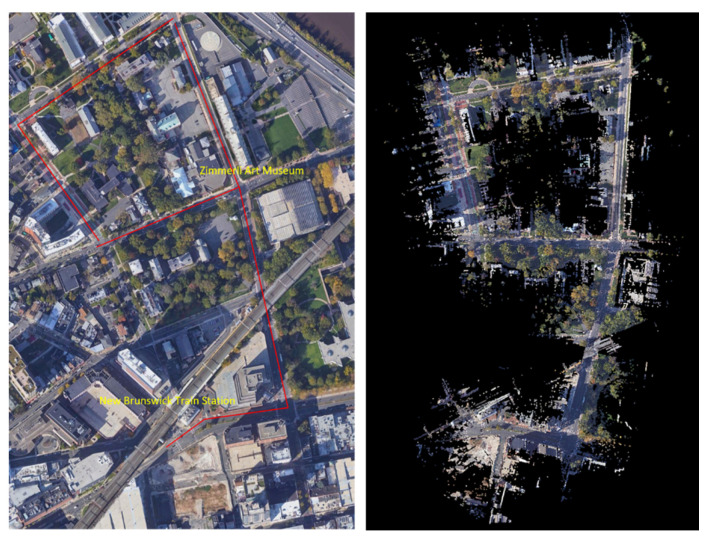
Evaluation Data Set ((**left**): Map data: Google, Maxar Technologies and (**right**): the mobile LiDAR data) along a route in downtown New Brunswick, NJ.

The mobile LiDAR system was used to map the entire downtown of New Brunswick, New Jersey. Part of the mobile LiDAR data set covering the routes connecting the New Brunswick train station to the Zimmerli Art Museum, as well as relevant satellite imagery, are used to evaluate various data representations and the SAM-based pedestrian infrastructure extraction approach (Figure 1). The route features multiple transportation modes, such as automobiles, bikes, e-scooters, and pedestrians, as well as complex intersection designs. Along the routes, there are Rutgers campus buildings, business establishments, hospitals, and many other types of amenities. The Zimmerli Art Museum also often hosts special events for people with disabilities, such as wheelchair users and people with visual impairment. All these facilities and activities lead to heavy use of pedestrian infrastructure along this route by all types of pedestrians. As a result, the data set features a high density of pedestrian infrastructure features, making it a complex and challenging one for machine-learning-based sidewalk inventory extraction. The mobile LiDAR data for the highlighted route are divided along the trajectory into 55 tiles at 500 MB per tile, covering a route length of approximately 1.5 km. The dataset, totaling 26 gigabytes, provides a comprehensive and high-resolution representation of the surveyed area. Segment Anything Model (SAM) has been designed to be promptable, and it can transfer zero-shot to new image distributions [23]. This paper focuses on evaluating SAM’s capability to segment a large number of pedestrian infrastructure features in various types of images generated from the point cloud and other data sources, such as satellite imagery.

### 3.2. SAM-Based Workflow

**Figure 2:**
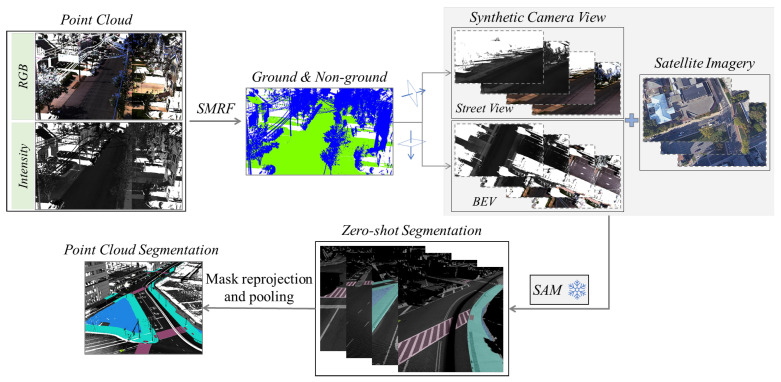
Elements of the proposed SAM-based multi-sourced pedestrian-accessible infrastructure segmentation workflow.

The SAM-based multi-sourced pedestrian-accessible infrastructure segmentation workflow includes the following three steps: (1) LiDAR data pre-processing; (2) Data projection and alignment; (3) Zero-shot segmentation; and (4) Reprojection and pooling of segmentation masks. Figure 2 illustrates the process. Large-scale mobile point-cloud data are segmented in the first step into ground and non-ground classes. Two different synthetic camera views (Street-view and BEV) are generated and aligned with the corresponding satellite image in the second step. In the third step, zero-shot segmentation is performed on the set of 2D images. Then, in the fourth step, object masks generated from the segmentation are reprojected back to the 3D geospatial space to segment the point cloud.

#### 3.2.1. Data Pre-Processing

The LiDAR point-cloud datasets were pre-processed with a common classification approach, which involves classifying the point cloud into classes that include ground, low vegetation, medium vegetation, and high vegetation. This approach starts with classifying ground points with the ground segmentation algorithms, namely Simple Morphological Filter (SMRF) [26]. Once the ground points are classified, heights from the ground are used to classify low vegetation, medium vegetation, and high vegetation. Also, in our paper, we projected a 3D point cloud onto 2D images using various methods: perspective projection for the street view and parallel projection for the bird’s eye view. Since these 2D images are generated using ideal imaging models, the alignment between the 3D point cloud and the pixels in the 2D BEV and street-view image is ensured. The satellite images are also geo-referenced with the point cloud, and the 2D geo-location of each pixel of satellite images can be matched with the x and y coordinates of the 3D points.

#### 3.2.2. Data Projection and Alignment

SAM is designed and trained to be promptable for image segmentation with strong zero-shot generalization [23]. To apply SAM to various modality data, i.e., 3D point clouds, we generate a stack of 2D images by projecting the point cloud from two different perspectives: Bird’s Eye View and Street View. The mobile LiDAR point-cloud data and satellite imagery are geo-referenced, and the extent of the point cloud is used to retrieve satellite imagery from Google. The specifics of data projection and alignment are elaborated upon in the subsequent paragraphs.

(1) Bird’s Eye View (BEV) representation

**Figure 3:**
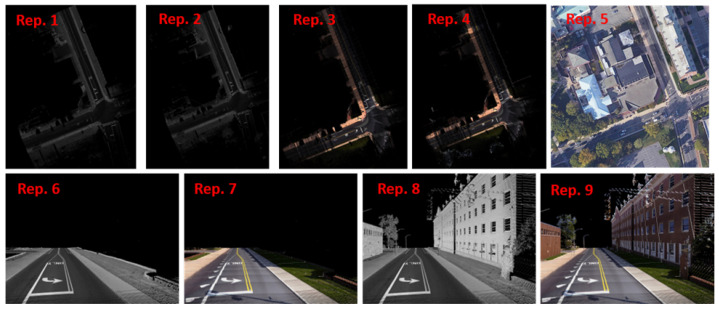
Image Representations: Rep. 1—BEV with only ground class and intensity values, Rep. 2—BEV with all classes and intensity values, Rep. 3—BEV with only ground class and color values, Rep. 4—BEV with all classes and color values, Rep. 5—Satellite imagery, Rep. 6—Street view with only ground class and intensity values, Rep. 7—Street view with only ground class and color values, Rep. 8—Street view with all classes and intensity values, and Rep. 9—Street view with all classes and color values.

The BEV representation is encoded by intensity (*I*) and color (RGB) through parallel projection [28]. We discretize the projected points into a 2D horizontal grid with a size of 0.1feet×0.1feet. The choice of this grid size consummates with the resolution of the point-cloud datasets. The BEV images are generated using the following Equation (1), where $BEV_{I}\left(i,j\right)$ and $BEV_{RGB}\left(i,j\right)$ is the value of pixel (i,j) generated using intensity and color, respectively, and $N_{ij}$ is the number of projected points in pixel (i,j). Four (4) variants of BEV images are generated by changing both the class of included points and the pixel attributes (Figure 3, Rep. 1 to Rep. 4). The possible values for the class of included points are with all classes and with only ground and low vegetation classes.

(1) $$BEV_{I}\left(i,j\right)=\frac{1}{N_{ij}}\sum_{k=0}^{N_{ij}}I_{k},BEV_{RGB}\left(i,j\right)=\frac{1}{N_{ij}}\sum_{k=0}^{N_{ij}}RGB_{k}$$

(2) Street-View representation

The street-view representation is generated using intensity (*I*) and color (RGB) information of the point cloud based on perspective projection [29]. The focal length *f* and pixel size ps of the virtual camera are set as 4.15 mm and 1.22 μm and the intrinsic matrix *A* of the virtual camera with a view size of 4032×3024 (width×height) is defined in Equation (2). For each position of the camera, *T*, an arbitrary camera orientation *R* can be defined; one front view and two side views are set to cover both the road and sidewalks in this study. The joint rotation-translation matrix R_T can be calculated using the camera position vector and orientation matrix. With customized camera matrix *A* and rotation-translation matrix R_T, virtual camera views are generated using the Open3D library based on Equation (3) [30].

(2) $$A=\left[\begin{array}{ccc} \frac{f}{ps} & 0 & \frac{width}{2}\\ 0 & \frac{f}{ps} & \frac{height}{2}\\ 0 & 0 & 1 \end{array}\right]$$

Given the mapping trajectory, the positions distancing equally sampled along the trajectory are used as the centers of the virtual camera locations for generating both BEV and street images. The distance between two camera locations should ensure that overlap exists between the synthetic views to cover all the ground points. The parameter “point size” is a critical factor that affects the final rendering results. We set the point size to 8 in the Open3D library. Similar to BEV images, four variants of street images for each snapshot are created by varying the class of points included and the pixel values (Figure 3, Rep. 6 to Rep. 9).

(3) $$s\left[\begin{array}{c} u\\ v\\ 1 \end{array}\right]=AR_{t}\left[\begin{array}{c} X\\ Y\\ Z\\ 1 \end{array}\right]$$

(3) Satellite Imagery

In the XYZ coordinate system of the LiDAR data, where z is the direction of the elevation, and the xy plane represents the ground. As the satellite imagery and the point cloud are geo-referenced, the x and y extents of each point cloud are used to fetch Google satellite imagery (illustrated in Figure 3, Rep. 5).

Figure 3 shows examples of a full stack of 2D view representations generated from a sample point-cloud file, plus a satellite image of the same scene. These images share the same geospatial coordinate system as the prime data source, i.e., the mobile point cloud. For the BEV and street-view images derived from the point cloud, all the pixels are aligned with the 3D point cloud based on the projection equations. The satellite images are also geo-referenced with the point cloud, and the 2D geo-location of each pixel of satellite images can be matched with *x* and *y* coordinates of the 3D points. In short, every segmentation mask generated by segmentation tools such as SAM on these images can be projected onto a planimetric map to create a comprehensive inventory of pedestrian infrastructure features.

#### 3.2.3. Zero-Shot Segmentation with a Web-Based Image Annotation Platform

**Figure 4:**
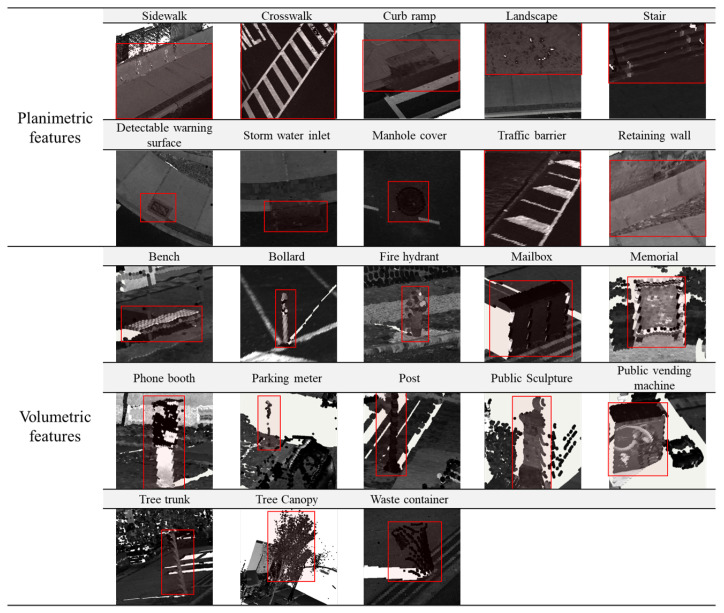
Examples of all the pedestrian-accessible infrastructures (labeled in red boxes) from 3D point-cloud space.

SAM is designed and trained to be promptable, and it can transfer zero-shot to novel images and object categories that have never been seen in its large segmentation training samples [23]. SAM consists of a powerful image encoder that can generate high-quality image embedding, and the features can then be queried by various prompts, e.g., points, box, mask, and text. Masks can be efficiently generated through a lightweight mask decoder after taking the image and prompt embedding. We deployed a SAM-based annotation project on CVAT, a web-based image annotation platform. In this web-based project, users can create different prompts, including point, box, and polygon, and then feed them to SAM for zero-shot image segmentation. From our practice, the point prompt requires less labor effort and can also generate high-quality masks of the target objects. A list of pedestrian infrastructure features is provided in Figure 4 to allow users to choose which pedestrian infrastructure object to segment. The object masks are archived and can be exported in common image annotation formats such as COCO 1.0 [31].

#### 3.2.4. Mask Reprojection and Pooling

In this project, the object masks are essentially polygons enclosing the boundaries of specific pedestrian infrastructure objects. The polygons are described in the image pixel coordinate system. Once zero-shot segmentation tasks are completed on a stack of images for the same area, we need to reproject the object masks back to the 3D geospatial space. This reprojection also enables the pooling of masks generated from multiple images to segment the original point cloud, allowing detailed geometric analysis, such as slope and grade assessment, on specific objects. Furthermore, it will facilitate the assembly of large 2D and 3D GIS maps.

### 3.3. Experiment and Assessment

**Figure 5:**
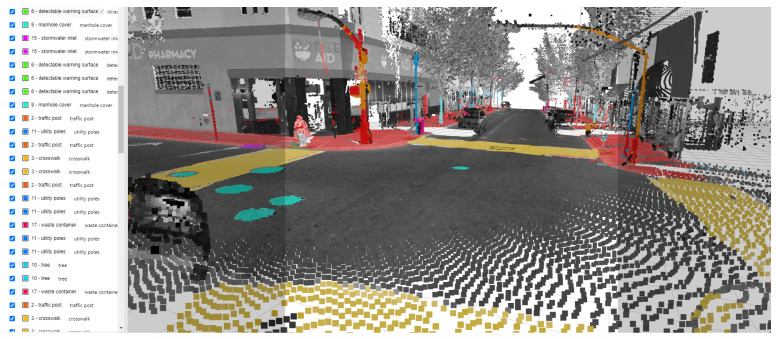
Pedestrian infrastructure features in a sample image.

One of the goals of this research is to identify data representations that can facilitate zero-shot segmentation with SAM. We evaluated the level of SAM-based pedestrian infrastructure extraction and segmentation of these infrastructure elements from these images of different representations and perspectives. A total of eight sets of images are used to test the performance of SAM with zero-shot learning. Each set of images includes 4 BEV images and 24 street-view images generated from mobile LiDAR point clouds, plus 1 satellite image. This brings the total number of images to 232. As indicated early in the paper, the entire data set covers a busy urban center, featuring a high density of pedestrian infrastructure features. For example, for a sample image as shown in Figure 5, there are 82 pedestrian infrastructure features to be segmented. The total number of pedestrian infrastructure features in these 232 images is more than 5000, a number far greater than the number of images. SAM was used to test its capability to segment these features correctly, not the images.

The choice of parameters, such as included classes of point clouds and types of pixel values, is driven by two considerations. First, including some classes of point clouds may present challenges rather than assistance in extracting pedestrian infrastructures. For example, points belonging to tree classes may occlude sidewalks. Second, The reflectance in various light spectrums serves as a valuable feature for distinguishing different types of pedestrian infrastructures. For example, color values are good for sidewalk segmentation, but intensity values are good for crosswalk detection.

The levels of zero-shot segmentation are expressed with no extraction (N), partial extraction (P), and complete extraction (C). If a particular type of pedestrian infrastructure object does not exist in the data set, an N/A was recorded. We also provide the reasons behind partial and no extraction in the hope of providing insights into why the extraction failed and, more importantly, on the possible ways of improving extractions with data fusion.

## 4. Results and Discussion

**Figure 6:**
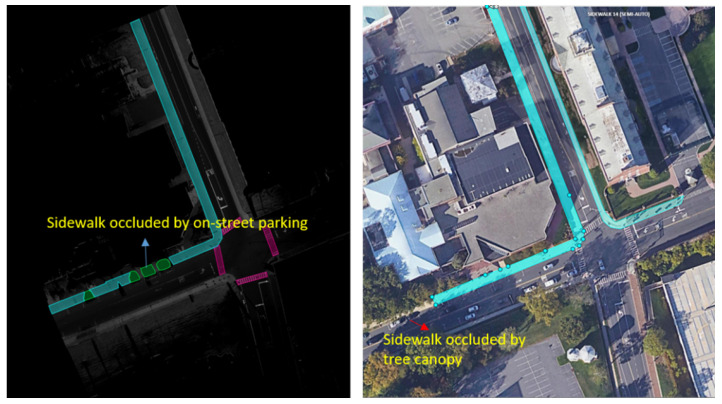
Sidewalk extraction with the fusion of mobile LiDAR and satellite imagery. The left image shows that the sidewalk occluded by on-street parking in the LiDAR data can be extracted in satellite imagery, while the right image shows that the sidewalk occluded by tree canopy in the satellite imagery can be extracted in the LiDAR data.

**Figure 7:**
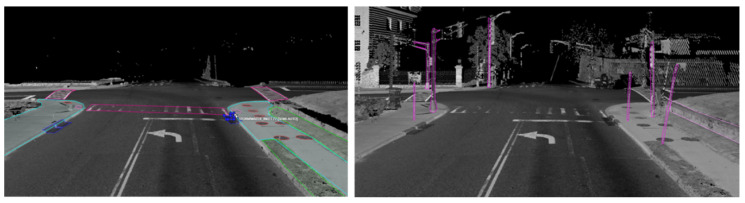
Segmentation of planimetric (**left**) and volumetric pedestrian infrastructure features (**right**) on street view images generated from point-cloud data.

**Figure 8:**
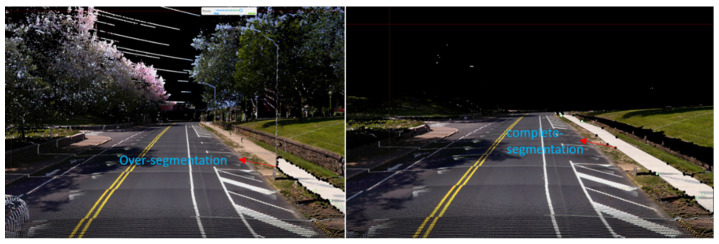
Over-segmentation on street view images with all classes.

Table 1 gives a snapshot of the evaluation results. The findings can be summarized as:

(1) ***Occlusions.*** Sidewalks can be severely occluded by tree canopies in satellite imagery, leading to incomplete segmentation of sidewalks. Occlusions to sidewalks by vehicles and other street objects are much less a concern when satellite imagery is used, but they limit what can be extracted from mobile LiDAR point clouds on busy street environments. On the other hand, mobile LiDAR data can capture data about sidewalks under tree canopies well. Therefore, combining mobile LiDAR data with satellite imagery can address each method’s weakness and allow for the complete extraction of sidewalks with SAM in most cases (Figure 6). However, we have found several pitfalls in using SAM. SAM-based zero-shot learning on our images was not stable, as SAM crashed multiple times. A considerable number of prompts, specifically clicks on areas in need of segmentation, are needed to achieve good segmentation. This is likely due to the size and resolution of these images, which are as large as 7000×10,000. In other words, SAM can work more effectively with satellite and BEV images with smaller fields of view or lower image resolutions. However, analyzing satellite imagery or BEV images at smaller patches may result in an impractical larger number of interactions and/or lower performance in segmentation.There is a need to automate the prompts by staging a proposal generation framework before SAM. This is one of our future research directions.
(2) ***Small volumetric features.*** It is generally difficult to extract small volumetric pedestrian infrastructure features, such as fire hydrants, parking meters, and posts, as well as small planimetric features, such as manhole covers, from either satellite imagery or BEV images generated from mobile LiDAR data. But they can be identified and segmented in high quality with SAM with very few prompts from street-view images that are generated from mobile LiDAR point-cloud data (Figure 7).
(3) ***Planimetric features.*** The SAM models achieved remarkable performance in segmenting planimetric pedestrian infrastructure features such as sidewalks, crosswalks, detectable warning surfaces, and curb ramps under zero-shot learning scenarios on street-view images that are generated from mobile LiDAR point clouds with only ground and low vegetation classes (Figure 7). Very few prompts are needed to achieve complete segmentation of these features. The exception cases are streets with significant amounts of street-level occlusions, such as on-street parking. On the other hand, over-segmentation of planimetric pedestrian infrastructure features can happen frequently in street-view images generated from mobile LiDAR point clouds with all classes (Figure 8).
(4) ***Color versus reflectance images.*** Segmenting pedestrian infrastructure objects in BEV and street-level images with color pixel values in some cases is easier than that of those intensity-based images as the color provides stronger clues about the boundary of these objects in the images. But this is only true in cases where the shadow is not of a concern. The intensity values, representing the strength of laser reflectance, are not influenced by shadows and light conditions. They are consistent for the same types of materials. In addition, the color information in Rep. 3, 4, 7, and 9 in Figure 3 are the results of data fusion, which involves aligning outputs of the laser scanners and cameras while the platform hosting them is moving at 10–40 miles per hour. The error in alignment is inevitable but will have significant impacts on the quality of data fusion.
(5) ***Street-view images.*** Street-level images with intensity values generated from mobile LiDAR data have the overall best performance in working with SAM to segment both planimetric and volumetric pedestrian infrastructure features. But if street-level images, those generated from mobile LiDAR data, are used as the sole source of data for extracting pedestrian infrastructure objects, we must address the street-level occlusion problem. One way to alleviate the problem is to conduct nighttime data collection, as laser scanning alone is not impacted by daylight, and there will be far fewer occlusions due to vehicles at night. In addition, this can be done through automated recognition of the presence of street occlusion objects and automated filling of occluded areas.

**Figure 9:**
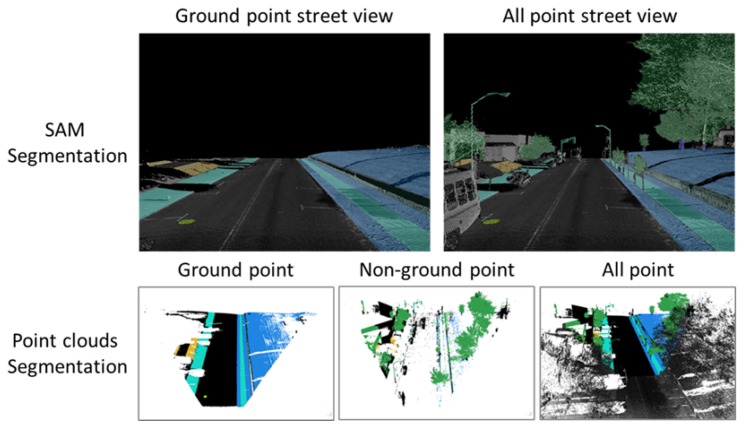
Example results of SAM-based workflow on mobile LiDAR point-cloud data.

**Figure 10:**
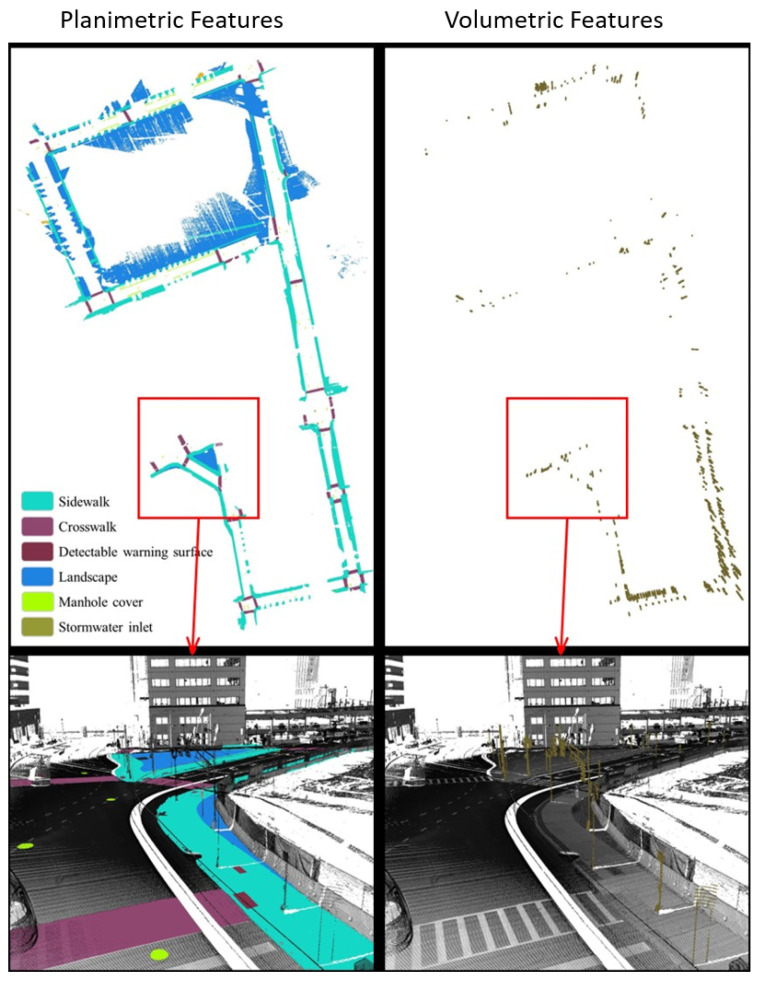
3D point-cloud semantic segmentation results. The left ground-based points mainly include sidewalks, crosswalks, detectable warning surfaces, landscape, manhole cover, and stormwater inlet. The pictures on the left show the volumetric features on the sidewalks, such as posts.

**Figure 11:**
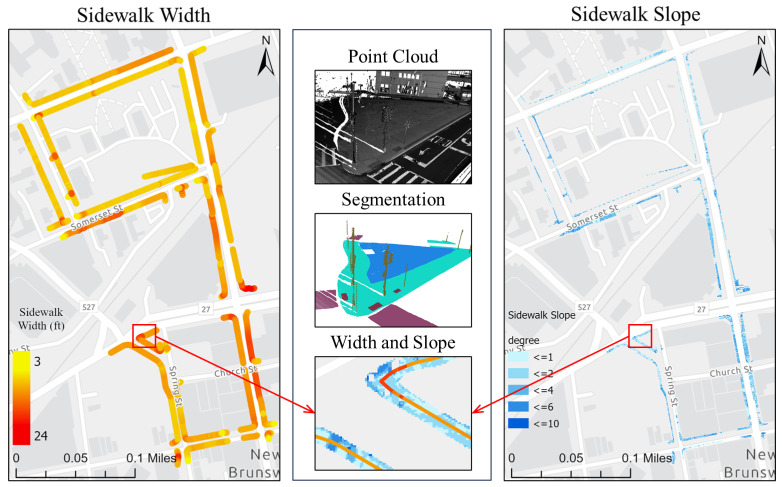
Computed widths and slopes for the entire network of sidewalks in the study area.

Overall, the SAM-based workflow can provide an end-to-end solution for GIS professionals to digitize pedestrian infrastructure objects with high accuracy. This is particularly true when the right image data representation is used (Figure 9). As part of the assessment, we did a complete extraction of the sidewalk features in the study area from the street views (Figure 10). These street views are intensity images from two types of point clouds, one with only the ground class and the other type with all the classes. Beyond showing the 2D locations of these pedestrian infrastructures, we further processed the segmented point clouds to characterize the sidewalk with important parameters, including width and slope (Figure 11). These results showed that the workflow by itself can already serve as a powerful tool for GIS professionals to process mobile LiDAR data into actionable information for improving pedestrian infrastructure.

## 5. Conclusions

In this paper, we designed and optimized a SAM-based pedestrian infrastructure segmentation workflow capable of efficiently processing multi-sourced geospatial data. We used an expanded definition of pedestrian infrastructure, termed as *pedestrian-accessible infrastructure*, which includes street furniture objects often omitted from the traditional definition. Our contributions lie in producing the necessary knowledge to answer the following two questions for pedestrian accessibility. First, which data representation can facilitate zero-shot segmentation of infrastructure objects with SAM? Second, how well does the SAM-based method perform in segmenting pedestrian infrastructure objects? Our findings indicate that street-view images generated from mobile LiDAR point-cloud data, when paired with satellite imagery data, can work efficiently with SAM to create a scalable pedestrian-accessible infrastructure inventory approach with immediate benefits to GIS professionals, transportation owners and walkers, especially those with travel-limiting disabilities. We demonstrated that the SAM-based workflow is capable of extracting sidewalk widths and slopes from the street-view images generated from mobile LiDAR data. This information is essential for individuals with visual and mobility impairments.

We would like to note that the scope of this paper is mostly limited to locating basic pedestrian-accessible infrastructure features. Additional processing is often needed to truly assess the compliance of these infrastructure features, especially those more complicated ones such as curb ramps. These additional processing steps will be included in our subsequent research. Our future research will also incorporate the training of deep learning models based on the annotated pedestrian infrastructure data to fully automate the entire workflow.

## Figures and Tables

**Table 1:** Evaluation results of different pedestrian infrastructure features using the SAM model. N: no extraction, P: partial extraction, C: complete extraction, and N/A: infrastructure type unavailable.

	Pedestrian Infrastructure Features	Rep. 1	Rep. 2	Rep. 3	Rep. 4	Rep. 5	Rep. 6	Rep. 7	Rep. 8	Rep. 9
Planimetric features	Sidewalk	P	P	P	P	P	C	C	C	C
	Crosswalk	C	C	C	C	C	C	C	C	C
	Curb ramp	P	P	P	P	N	C	C	C	C
	Landscape	P	P	P	P	P	C	C	P	P
	Stair	N	N	P	P	N	N	N	C	C
	Detectable warning surface	N	C	N	C	N	C	C	C	C
	Storm water inlet	N	N	N	N	N	C	C	C	C
	Manhole cover	N	N	N	N	N	C	C	C	C
	Traffic barrier	N	N	N	N	N	N	N	C	C
	Retaining wall	N	N	N	N	N	C	C	C	C
Volumetric features	Bench	N/A	N/A	N/A	N/A	N/A	N/A	N/A	N/A	N/A
	Bollard	N/A	N/A	N/A	N/A	N/A	N/A	N/A	N/A	N/A
	Fire hydrant	N	N	N	N	N	N	N	C	C
	Mailbox	N/A	N/A	N/A	N/A	N/A	N/A	N/A	N/A	N/A
	Memorial	N/A	N/A	N/A	N/A	N/A	N/A	N/A	N/A	N/A
	Phone booth	N/A	N/A	N/A	N/A	N/A	N/A	N/A	N/A	N/A
	Parking meter	N	N	N	N	N	C	C	C	C
	Post	N	N	N	N	N	N	N	C	C
	Public Sculpture	N/A	N/A	N/A	N/A	N/A	N/A	N/A	N/A	N/A
	Public vending machine	N/A	N/A	N/A	N/A	N/A	N/A	N/A	N/A	N/A
	Tree trunk	N	N	N	N	N	N	N	C	C
	Tree Canopy	N	N	P	P	P	N	N	P	P
	Waste container	N	N	N	N	N	N	N	C	C

## Data Availability

The data presented in this study are available upon request.
